# PLCE1 Polymorphism and Upper Gastrointestinal Cancer Risk: A Meta-Analysis

**DOI:** 10.1371/journal.pone.0067229

**Published:** 2013-06-24

**Authors:** Ning-Bo Hao, Ya-Fei He, Dan Zhang, Gang Luo, Bai-Jun Chen, Yao Zhang, Shi-Ming Yang

**Affiliations:** 1 Department of Gastroenterology, Xinqiao Hospital, Third Military Medical University, Chongqing, China; 2 Department of Epidemiology, Third Military Medical University, Chongqing, China; 3 The Evidence Based Medicine and Clinic Epidemiology Center, Third Military Medical University, Chongqing, China; 4 Chongqing Key Laboratory for Diseases Proteomics, Southwest Hospital, Third Military Medical University, Chongqing, China; Tongji Medical College, Huazhong University of Science and Technology, China

## Abstract

**Background:**

In recent years, the PLCE1 rs2274223 polymorphism has been extensively investigated as a potential risk factor for upper gastrointestinal cancers, including squamous cell carcinoma (ESCC) and gastric cancer. However, the results of these studies have been inconsistent.

**Methods:**

A meta-analysis of 13 case-control studies was performed including more than 11,000 subjects with genotyped PLCE1 rs2274223 polymorphisms. Odds ratios (OR) with 95% confidence intervals (CI) were employed to assess the association of the PLCE1 rs2274223 polymorphism with a susceptibility to ESCC or gastric cancer.

**Results:**

A statistically significant increase in the risk of ESCC was associated with the PLCE1 rs2274223 polymorphism. This included the homozygous genetic model (OR = 1.46), heterozygous genetic model (OR = 1.25) and allelic genetic model (OR = 1.23). Similar results were consistently found for gastric cancer. In a subgroup analysis, the PLCE1 rs2274223 polymorphism was found to be a very sensitive marker for gastric cardia cancer as shown by the homozygous genetic model (OR = 2.23), heterozygous genetic model(OR = 1.59) and allelic genetic model (OR = 1.47). The risk associations of all of the gastric cardia cancer models were statistically significant. In contrast, none of the genetic models for non-cardia gastric cancer were significant.

**Conclusions:**

In this meta-analysis, the PLCE1 rs2274223 polymorphism was confirmed to have a statistically significant association with an increasing risk of ESCC and gastric cancer. The increase risk was especially observed for gastric cardia cancer.

## Introduction

Cancer is the leading cause of death in both developed and developing countries. Recent research has estimated that approximately 12.7 million cancer cases and 7.6 million cancer deaths have occurred worldwide. Among these, 738,000 and 406,800 deaths were caused by esophageal and gastric cancer, respectively. Accordingly, esophageal cancer corresponds to the second leading cause and gastric cancer corresponds to the sixth leading cause of cancer-related deaths worldwide [1]. The incidence rates of esophageal and gastric cancer vary internationally. The highest rates of esophageal cancer are found in Southern and Eastern Africa and Eastern Asia. In these areas, 90% of the cases are esophageal squamous cell carcinomas (ESCCs) [2]. Major risk factors of esophageal cancer are thought to include poor nutritional status, low intake of fruits and vegetables, and consumption of high temperature beverages [3–5]. The highest incidence rates for gastric cancer are in Eastern Asia, Eastern Europe, and South America [1]. *Helicobacter pylori* is considered the main etiologic factor in all populations, although most infected individuals do not develop cancer [6]. However, the nature of this mechanism is not fully understood. In recent years, genetic predisposition has been considered to be a combined influence factor. Gene Wide Association Studies (GWAS) play important roles in the identification of potential candidates for single nucleotide polymorphisms (SNPs).

Phospholipase C epsilon 1 (PLCE1), which is located on chromosome 10q23, encodes a phospholipase that hydrolyzes phosphatidyl-inositol 4,5-bisphosphate to 1,2-diacylglycerol and inositol 1,4,5-trisphosphate [7]. This phospholipase has been reported to be associated with intracellular signaling through the regulation of a variety of proteins such as the protein kinase C (PKC) isozymes and the proto-oncogene ras [8,9]. As early as 2006, Hinkes et al. found that PLCE1 gene variants cause an early onset nephrotic syndrome in humans [10]. Thereafter, it was demonstrated that PLCE1 is also associated with carcinogenesis, including cancers of the intestine, bladder, skin, colon, rectum, head and neck [11–15]. In 2010, two large-scale GWASs simultaneously reported that a new susceptibility locus (rs2274223: A5780G), located in exon 26 of PLCE1, was strongly associated with the risk of ESCC and gastric cancer in Chinese populations [16,17]. However, recent studies in this area have produced inconsistent results. Both Plamer et al. and Dura et al. found that the rs2274223 polymorphism in PLCE1 was associated with a reduced risk of ESCC. Further, no association with gastric cancer was found in either a Polish or a US study [18,19]

In this study, a systematic review and meta-analysis were performed to clarify these inconsistencies and to establish a comprehensive picture of the relationship between PLCE1 gene variants and the risk of ESCC and gastric cancer. In addition, recent studies reported that a series of gene mutation such as adenosine diphosphate ribosyltransferase (ADPRT) and X-ray repair cross-complementing 1 (XRCC1), matrix metalloproteinases 2 (MMP-2), and cyclooxygenase-2 (COX-2) were only associated with the gastric cardia cancer [20–22]. So in this article we also made a stratified analysis and found that PLCE1 polymorphism is significantly associated with gastric cardia cancer but not with gastric non-cardia cancer.

## Materials and Methods

### Literature sources and search strategies

This meta-analysis were performed by searching PubMed, MEDLINE, EMBASE, Web of Science databases, Cochrane Library and the Chinese Biomedical Literature database (CBM) (updated to December, 2012). No language restriction was set. The combination of key words was as follows: ‘PLCE1 polymorphism’, ‘rs2274223’, ‘esophageal squamous cell carcinoma’ or ‘ESCC’ and ‘gastric cancer’. All eligible articles were screened, and their references were checked for other relevant studies. Our study was conducted in accordance with the standard for meta-analysis of observational studies in epidemiology.

#### Inclusion and exclusion criteria

The studies were selected based on the following inclusion criteria: 1) the studies evaluated the PLCE1 polymorphism and the risk of ESCC or gastric cancer; 2) all patients were histo-pathologically diagnosed as primary ESCC or gastric cancer; 3) the studies were case-control design; 4) the genotype distributions for both the cases and the controls were available for estimating odds ratios (OR) and 95% conﬁdence intervals (CI); and 5) the studies met the assumptions for Hardy-Weinberg equilibrium (HWE). In addition, the following exclusion criteria were used: 1) the studies did not provide detailed data such as presented in abstracts, meeting reports and reviews; 2) the genotype frequency was not reported; 3) the studies were repeated or overlapped other publications; and 4) the controls do not meet the assumptions for HWE.

### Data extraction

The data were obtained according to a standard protocol. All of the studies included in this meta-analysis meet the inclusion and exclusion criteria. The accuracy of the extracted information was assured by the extraction of the raw data by two independent researchers (Hao and He). Evaluation conflicts were resolved by a discussion among all of the investigators. The collected data comprised the name of the first author, the year of publication, the regions where the studies were conducted, the total number of case and control subjects, the number of genotypes examined, the genotyping results, the matching criteria and the HWE.

### Statistical analyses

In the current meta-analysis, homozygous (GG *vs* AA), heterozygous (GA *vs* AA) and allelic (G vs A) models were used. All analyses were performed with STATA 11 (Stata Corp LP, College Station, TX). The ORs and their corresponding 95% CIs were used to evaluate the association between the PLCE1 polymorphisms and the risk of ESCC or gastric cancer. The statistical signiﬁcance of the summary OR was determined by the Z test, and a P value less than 0.05 was considered statistically significant. Heterogeneity was determined by the Chi-squared-based Q-test with the significance level set at a P value less than 0.10. If there was heterogeneity, the ORs were calculated with the random effects model [23]. Otherwise, calculations were performed with the fixed effects model [24]. HWE was accessed using Fisher’s exact test with the significance set at a P value less than 0.05.A sensitivity analysis was performed to assess the stability of the results. Publication bias was analyzed by the use of Begg’s test and Egger’s test [25,26].

## Results

### Study inclusion and characteristics

**Figure 1:**
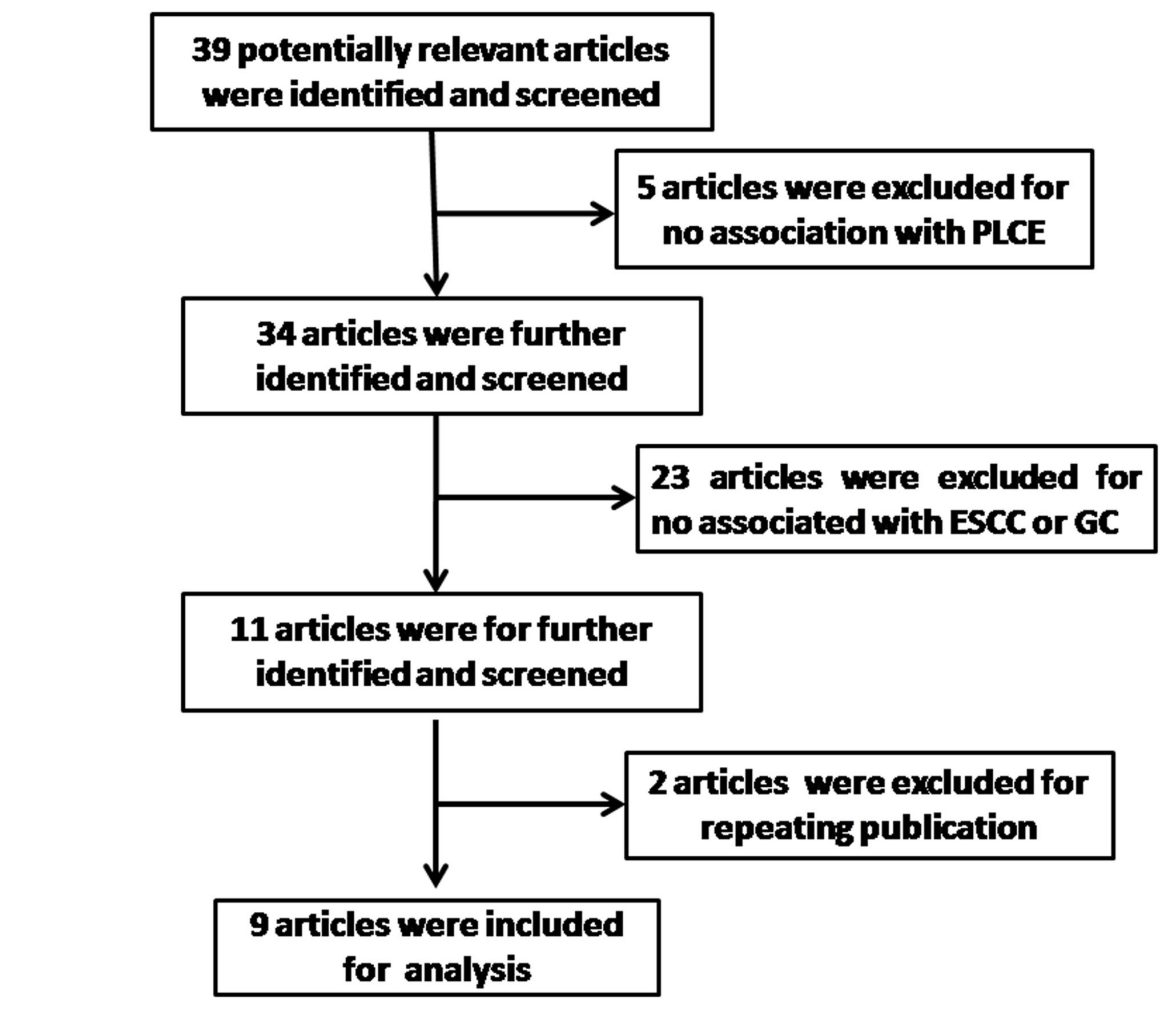
Flow of study identification, inclusion, and exclusion.

The combined search yielded 93 references. The study selection process is shown in Figure 1.A total of thirteen studies from nine articles were ultimately included; this resulted in the examination of 10,353 patients and 13,902 controls [16–19,27–31]. Eight studies were available for ESCC, and this included a total of 5,226 cases and 8,111 controls. Five studies, with 5,127 cases and 5,791 controls, met the inclusion/exclusion criteria for gastric cancer. All of the studies included in the meta-analysis were consistent with HWE in the control populations. The detailed characteristics of the studies included in this meta-analysis are shown in Table 1. There are four studies of Caucasians, seven studies of Chinese, one study of black and one of mixed ancestry.

**Table 1:** Characteristics of studies included in meta-analysis.

**Study**	**Years**	**Ethnicity**	**No. of Case/Control**	**Matching Criteria**	**Control Source**	**Genotyping**	**HWE**
**Esophageal squamous cell carcinoma**							
Abnet	2010	Chinese	1898/2100	Sex, Age,	PB	TaqMan	Yes
Palmer	2012	Caucasian(US)	52/410	Sex, Age,	PB	PCR	Yes
Wang	2010	Chinese	1077/1733	Sex, Age	HB	TaqMan	Yes
Hu	2012	Chinese	1061/1211	Sex, Age	HB	TaqMan	Yes
Gu	2012	Chinese	380/380	Sex, Age	HB	MALDI-ToF–MS	Yes
Bye	2012	Black	418/850	Sex, Age	PB	TaqMan	Yes
Bye	2012	Mixed Ancestry	254/847	Sex, Age	PB	TaqMan	Yes
Dura	2012	Caucasian	86/580	Sex, Age	PB	PCR	Yes
**Gastric cancer**							
Abnet	2010	Chinese	1625/2100	Sex, Age,	PB	TaqMan	Yes
Zhang	2011	Chinese	1848/1665	Sex, Age	PB	PCR	Yes
Palmer	2012	Caucasian (Polish)	289/376	Sex, Age,	PB	PCR	Yes
Palmer	2012	Caucasian (US)	306/420	Sex, Age,	PB	PCR	Yes
Wang	2012	Chinese	1059/1240	Sex, Age,	PB	TaqMan	Yes

NA: not available; PB: population based; HB hospital based; MALDI-ToF–MS: matrix-assisted laser desorption/ionization time-of-ﬂight mass spectrometry

### Pooled analyses

**Figure 2:**
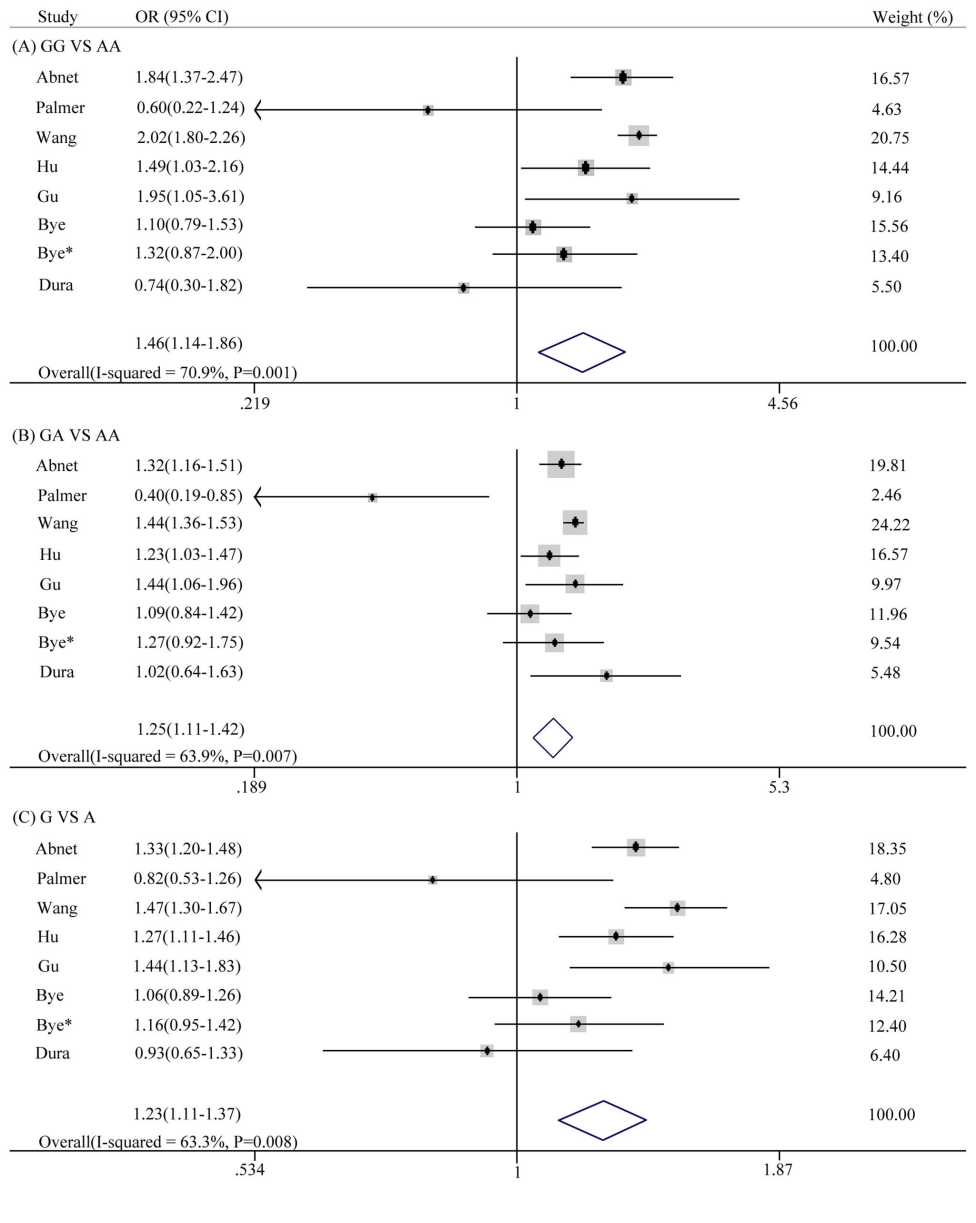
Forest plot of the ESCC risk associated with the PLCE1 rs2274223 polymorphism. (A) GG *vs* AA, (B) GA *vs* AA, (C) G *vs* A.

Eight studies examining ESCC were included for the evaluation of the association with the PLCE1 rs2274223 polymorphism. Because the heterogeneity was almost obvious (all P values < 0.05), the random effects model was used. A significantly increased ESCC risk was found in all of the genetic models, including the homozygous genetic model (GG *vs* AA: OR = 1.46, 95% CI: 1.14-1.86, P = 0.002), the heterozygous genetic model (GA *vs* AA: OR = 1.25, 95% CI: 1.11-1.42, p = 0.000) and the allelic genetic model (G *vs* A: OR = 1.23, 95% CI: 1.11-1.37, p = 0.000) (Figure 2).

**Figure 3:**
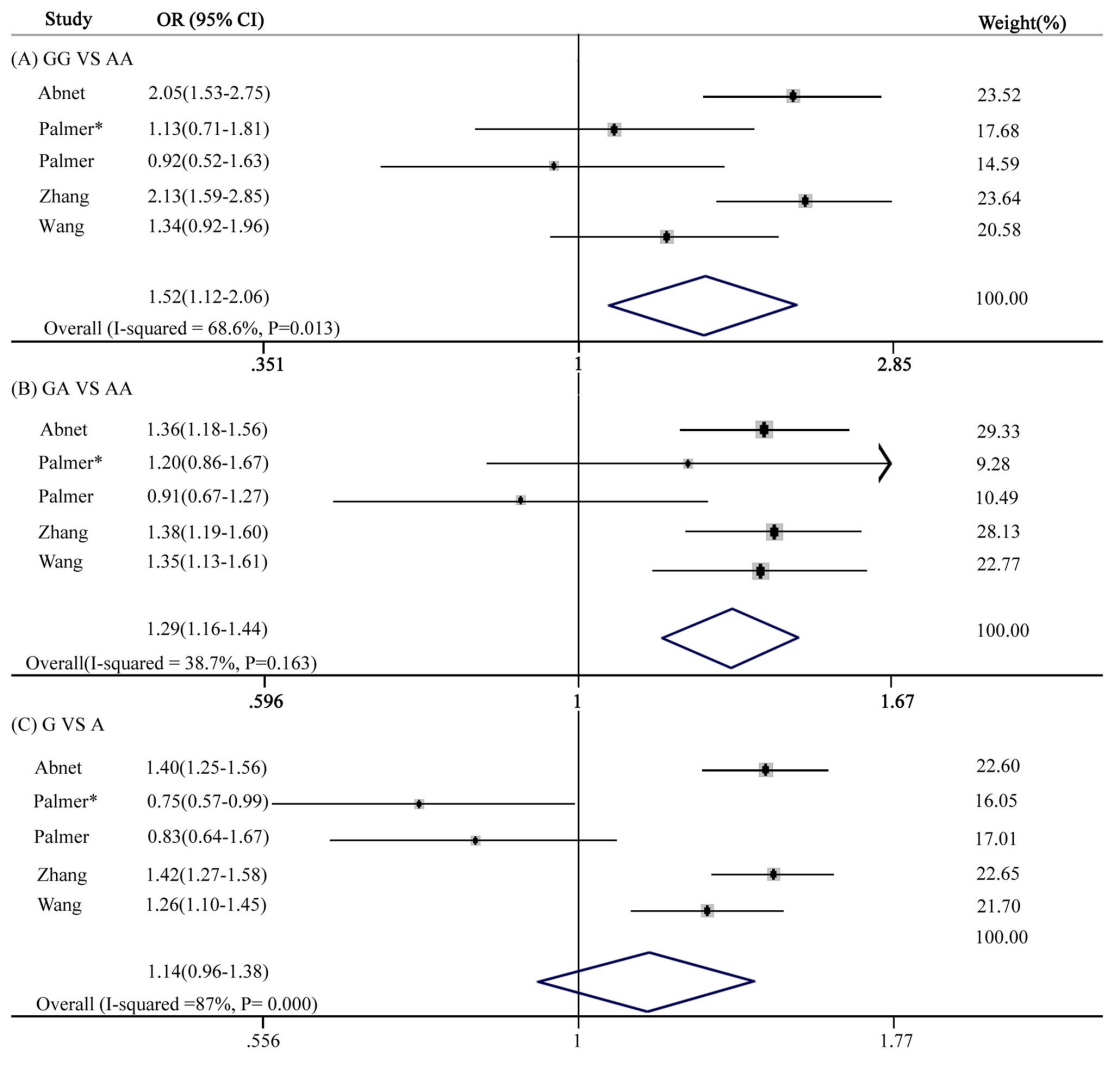
Forest plot of the gastric cancer risk associated with the PLCE1 rs2274223 polymorphism. (A) GG *vs* AA, (B) GA *vs* AA, (C) G *vs* A.

Five gastric cancer studies were analyzed using the random effects model because heterogeneity was obvious. Statistically significant associations were found between the occurrence of the rs2274223 polymorphism and increased gastric cancer risk under the homozygous genetic model (GG *vs.* AA: OR = 1.52, 95% CI: 1.12-2.06, p = 0.007), and the heterozygous genetic model (GA *vs.* AA: OR = 1.29, 95% CI: 1.16-1.44, p = 0.000). However, a statistically significant association was not found under the allelic genetic model (G *vs.* A: OR = 1.14, 95% CI: 0.94-1.38, p = 0.193) (Figure 3).

**Figure 4:**
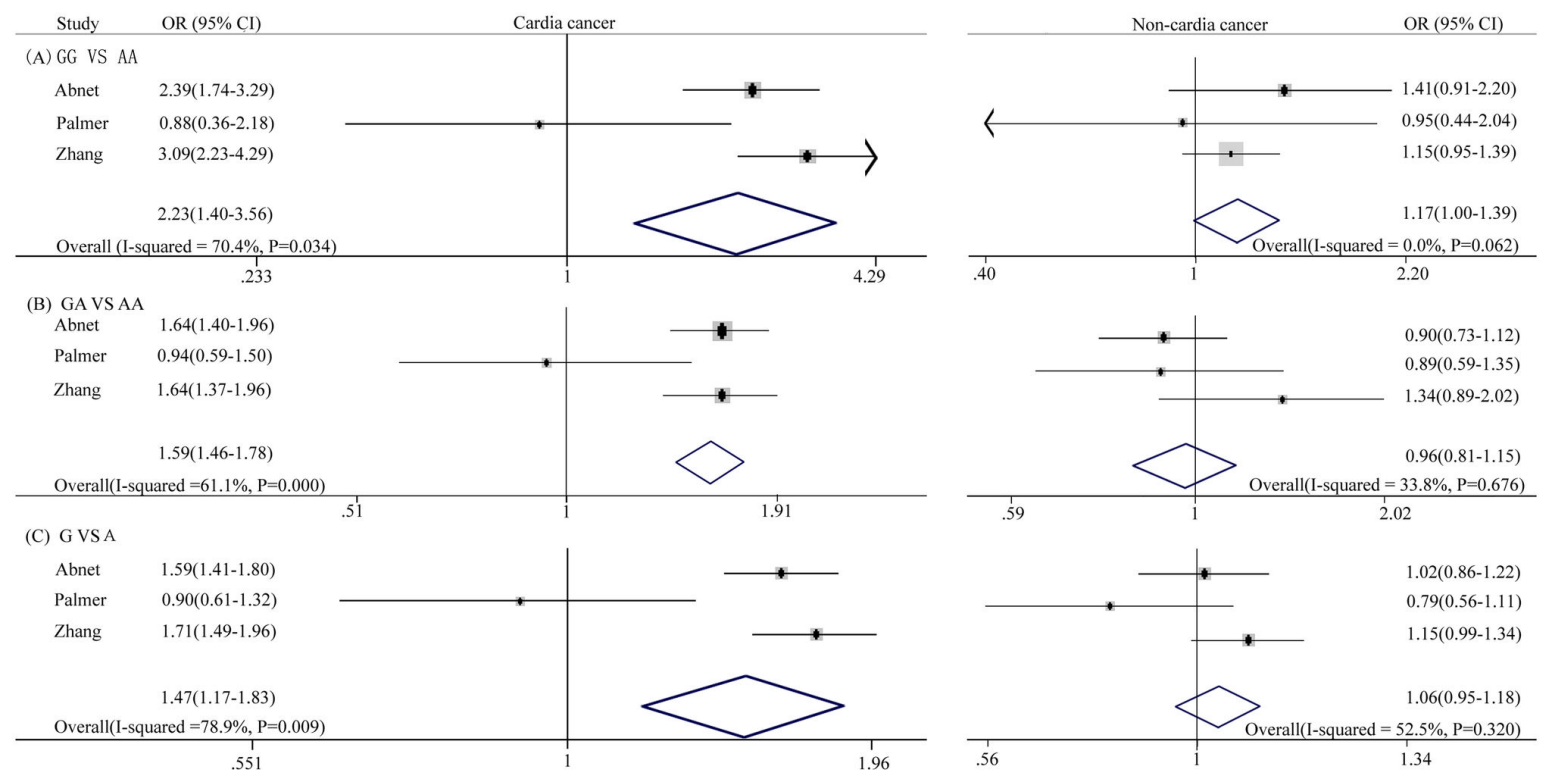
Forest plot of gastric cardia cancer and non-cardia gastric cancer risk associated with PLCE1 rs2274223 polymorphism. (A) GG *vs* AA, (B) GA *vs* AA, (C) G *vs* A.

In addition, three studies divided gastric cancer into the gastric cardia cancer and the non-cardia gastric cancer subtypes. Consequently, analyses were performed examining the association between the rs2274223 polymorphism and the risk of both gastric cardia cancer and non-cardia gastric cancer. For gastric cardia cancer, the random effects model was used. A statistically significant increase in the risk of gastric cardia cancer was found in all the genetic models. This includes the homozygous genetic model (GG *vs.* AA: OR = 2.23, 95% CI: 1.40-3.56, p = 0.001), the heterozygous genetic model (GA *vs.* AA: OR = 1.59, 95% CI: 1.42-1.78, p = 0.000) and the allelic genetic model (G *vs.* A: OR = 1.47, 95% CI: 1.17-1.83, p = 0.001). The evaluation of non-cardia gastric cancer used fixed effects models. The results of the meta-analysis result showed that the rs2274223 polymorphism genotype might have an association with decreased non-cardia gastric cancer risk. This included the homozygous genetic model (GG *vs.* AA: OR = 1.17, 95% CI: 0.99-1.39, p = 0.001), the heterozygous genetic model (GA *vs.* AA: OR = 0.96, 95% CI: 0.81-1.15, p = 0.676) and the allelic genetic model (G *vs.* A: OR = 1.06, 95% CI: 0.95-1.18, p = 0.320) (Figure 4).

### Sensitivity analyses and publication bias

Sensitivity analyses were performed to assess the inﬂuence of each individual study on the pooled ORs by the systematic omission of the individual studies from the analyses. The corresponding pooled ORs were not materially altered for all the subject and subgroup examinations of PLCE1 genotypes (data not shown). Begg’s funnel plot and Egger’s linear regression test were performed to assess the publication bias of included studies. The shape of the funnel plots was symmetrical for the two polymorphisms (data not shown). The statistical results did not show a publication bias in these studies.

## Discussion

PLCE1 is a member of the phospholipase C family of proteins. It differs from other family members and can uniquely interact with the Ras proto-oncogene. PLCE1 also acts as an effector of guanosine triphosphatases (Ras, Rap1 and Rap2), which are involved in the regulation of cell growth, differentiation, apoptosis and angiogenesis [9,15,32]. Because mutations in the RAS gene family are associated with approximately 30% of all human cancers, several studies have investigated the possible role of PLCE1 mutations in cancer development and progression [12–15,33]. PLCE1 has been reported to have multiple mutation points such as rs2274223, rs11187870 and rs3765524 [17,19,28]. All these studies discussed the relationship between the rs2274223 gene variant and the ESCC or gastric cancer risk. However, the rs11187870 and rs3765524 gene variants have been studied less. This meta-analysis only examines the association between the rs2274223 gene variant and the ESCC and gastric cancer risk.

PLCE1 rs2274223 is a non-synonymous SNP with an A to G transition in exon 26, which results in the substitution of a histidine for an arginine. In this meta-analysis, we confirmed that PLCE1 rs2274223 was associated with an increase in the risk of ESCC under all the genetic models including the homozygous genetic model (OR = 1.46), heterozygous genetic model (OR = 1.25) and allelic genetic model (OR = 1.23). Furthermore, it was also demonstrated that PLCE1 rs2274223 was a notable signal for susceptibility to gastric cancer under the homozygous genetic model (OR = 1.52) and the heterozygous genetic model (OR = 1.29). However, no significant association was found for the allelic genetic model (OR= 1.14). It appears that PLCE1 may not be a risk factor for all gastric cancers. When gastric cancer was divided into the gastric cardia cancer and the non-cardia gastric cancer subtypes, the results showed a significant association for gastric cardia cancer in all the genetic models. This included the homozygous genetic model (OR = 2.23), the heterozygous genetic model (OR = 1.59) and the allelic genetic model (OR = 1.47). In contrast, we found no association with non-cardia gastric cancer for any of the genetic models.

This meta-analysis confirmed that PLCE1 rs2274223 was associated with an increase in the risks of ESCC and gastric cancer, especially gastric cardia cancer. Recently, another study found that PLCE1 rs2274223 was associated with survival following a gastric cancer diagnosis. These investigators examined a total of 938 gastric cancer patients and found that individuals carrying the PLCE1 rs2274223 AG/GG genotype had a higher survival rate than those carrying the AA genotype. This suggested that the rs2274223 G allele might be associated with better prognosis in gastric cancer patients [34]. It appears that although PLCE1 rs2274223 was associated with a high risk of gastric cancer, carriers of this genotypealso had a long survival. However, the reason for this effect of the PLCE1 rs2274223 gene polymorphism is still unclear. Wang et al. found that the rs2274223 gene variant was more evident in males, non-smokers, non-drinkers and gastric cardia cancer patients [30].

In interpreting the results of this study, some limitations of the meta-analysis should be addressed. First, a portion of the controls may have been exposed to unknown bias factors. This is because they were hospital based, which could affect the reliability of the meta-analysis. However, the hospital-based controls were only used by a small number of the studies. Second, the lack of individual-level data prevented further analyses to identify interactions between the genetic variations and the metabolic traits. Third, only published studies were included in this meta-analysis. Therefore, a publication bias may have occurred, even though such a bias was not revealed by the statistical testing.

In conclusion, this meta-analysis indicated that the PLCE1 rs2274223 polymorphism is associated with both ESCC and gastric cancer susceptibility. In addition, this polymorphism could possibly serve as a biomarker for cancer risk. This meta-analysis found that the PLCE1 rs2274223 gene variant was linked to greater susceptibility for gastric cardia cancer. However, this effect was not evident for non-cardia gastric cancer.

## Supporting Information

File S1 PRISMA Checklist. (DOC)
